# Amygdalin Alleviates DSS-Induced Colitis by Restricting Cell Death and Inflammatory Response, Maintaining the Intestinal Barrier, and Modulating Intestinal Flora

**DOI:** 10.3390/cells13050444

**Published:** 2024-03-03

**Authors:** Dianwen Xu, Yachun Xie, Ji Cheng, Dewei He, Juxiong Liu, Shoupeng Fu, Guiqiu Hu

**Affiliations:** 1 State Key Laboratory for Diagnosis and Treatment of Severe Zoonotic Infectious Diseases, Key Laboratory for Zoonosis Research of the Ministry of Education, Institute of Zoonosis and College of Veterinary Medicine, Jilin University, Changchun 130062, China; xudw21@mails.jlu.edu.cn (D.X.); xieyachun2022@163.com (Y.X.); chengji21@mails.jlu.edu.cn (J.C.); juxiong@jlu.edu.cn (J.L.); 2 College of Animal Science, Jilin University, Changchun 130062, China; m13144303829@163.com

**Keywords:** amygdalin, colitis, intestinal barrier, apoptosis and ferroptosis, intestinal flora

## Abstract

Inflammatory bowel disease (IBD) refers to a cluster of intractable gastrointestinal disorders with an undetermined etiology and a lack of effective therapeutic agents. Amygdalin (Amy) is a glycoside extracted from the seeds of apricot and other *Rosaceae* plants and it exhibits a wide range of pharmacological properties. Here, the effects and mechanisms of Amy on colitis were examined via 16S rRNA sequencing, ELISA, transmission electron microscopy, Western blot, and immunofluorescence. The results showed that Amy administration remarkably attenuated the signs of colitis (reduced body weight, increased disease activity index, and shortened colon length) and histopathological damage in dextran sodium sulfate (DSS)-challenged mice. Further studies revealed that Amy administration significantly diminished DSS-triggered gut barrier dysfunction by lowering pro-inflammatory mediator levels, inhibiting oxidative stress, and reducing intestinal epithelial apoptosis and ferroptosis. Notably, Amy administration remarkably lowered DSS-triggered TLR4 expression and the phosphorylation of proteins related to the NF-κB and MAPK pathways. Furthermore, Amy administration modulated the balance of intestinal flora, including a selective rise in the abundance of *S24-7* and a decline in the abundance of *Allobaculum*, *Oscillospira*, *Bacteroides*, *Sutterella*, and *Shigella*. In conclusion, Amy can alleviate colitis, which provides data to support the utility of Amy in combating IBD.

## 1. Introduction

Inflammatory bowel disease (IBD) is a digestive disorder that is categorized into ulcerative colitis (UC) and Crohn’s disease (CD) [1]. Its pathological characteristics primarily comprise chronic intestinal inflammation, gut epithelial dysfunction, and mucosal damage of the gastrointestinal tract, which can cause clinical symptoms such as abdominal pain, diarrhea, mucopurulent bloody feces, and weight reduction [2]. Moreover, IBD, especially UC, represents a major causative factor in colorectal cancer (CRC) if left untreated [3]. Even now, no cure for IBD exists and its clinical treatment aims to control clinical symptoms, relieve intestinal inflammation, promote mucosal healing, and reduce recurrence. Current drugs used to treat IBD include corticosteroids, 5-aminosalicylic acid, etc. However, the long-term application of these drugs is prone to side effects and does not achieve the desired effect [4]. Therefore, an effective and safe way to prevent and alleviate IBD is urgently needed.

Biologically active substances from natural plants, mostly with low toxicity and adaptability, are effective in preventing and alleviating IBD [5,6]. Amygdalin (Amy), also referred to as vitamin B17, is a natural hydroxyglycoside taken from the seeds of plants in the *Rosaceae* family (e.g., apples, peaches, cherries, apricots, and plums) [7]. It is gaining interest for its pharmaceutical potential [8], and is used to treat asthma, bronchitis, emphysema, vitiligo, leprosy, diabetes, and cancer [9]. Studies have demonstrated that *Mume Fructus* and *Prunus mume* fruit containing Amy exert protective effects against 2,4,6-trinitrobenzenesulphonic acid (TNBS)-induced colitis in rats [10]. Moreover, a very recent article showed that essential oil from *Pruni Semen* (PSEO) has a protective effect against DSS-induced ulcerative colitis in mice and that its active ingredients are mainly products of Amy hydrolysis [11]. However, the potential role and mechanism of Amy as a monomer in IBD is unknown and deserves further investigation.

Although the pathogenesis of IBD has not been fully understood, inflammatory response, oxidative stress, intestinal barrier dysfunction, and excessive intestinal epithelial cell death have been shown to be involved [12,13]. Meanwhile, Amy showed good properties in mitigating inflammation, oxidative stress, and cell death. For example, Amy significantly reduced the expression of cytokines and inflammatory cell infiltration in lipopolysaccharide (LPS)-induced acute lung injury [14]. Amy attenuated D-galactosamine- and LPS-induced acute liver injury by modulating NLRP3, NF-κB, and nuclear factor erythroid 2-related factor 2 (Nrf2) signaling pathways [15]. Moreover, Amy can reduce cell death in non-cancer cells. For example, Amy exerted anti-apoptotic effects in LPS-stimulated BEAS-2B bronchial epithelial cells [16] and inhibited inflammation and apoptosis in ox-LDL-treated bone-marrow-derived macrophages and ApoE knock-out mice [17]. Amy also blocked PM2.5-triggered inflammation, oxidative stress, and apoptosis in HUVECs [18]. Importantly, one study found that Amy attenuated inflammation and oxidative stress by increasing the expression of glutathione peroxidase 4 (GPX-4) and Nrf2 [19], which reflects its potential association with ferroptosis. Therefore, we speculate that Amy may have good therapeutic effects on IBD.

Furthermore, the research into humans and mice shows the involvement of intestinal flora in the maintenance of intestinal homeostasis and the pathophysiology of multiple diseases, including IBD and CRC [20]. Disturbances in the intestinal flora are closely associated with intestinal mucosal damage and dysfunction of the epithelial barrier [21]. Therefore, the regulation of intestinal dysbiosis is considered to be an important target for the treatment of colitis. For example, Acteoside and Parthenolide alleviated colitis by remodeling the intestinal flora in dextran sodium sulfate (DSS)-treated mice [22,23]. However, whether Amy has a modulating effect on intestinal flora in DSS-treated mice remains to be investigated.

Therefore, the present study aimed to investigate the protective effects of Amy against UC and its mechanisms by establishing a DSS-induced mouse model of UC, highlighting its modulatory effects on inflammation, oxidative stress, cell death, and the intestinal barrier. In addition, the regulatory effects of Amy on the intestinal flora of DSS-treated mice were revealed via intestinal flora sequencing.

## 2. Materials and Methods

### 2.1. Materials

Amygdalin (Amy, ≥98%) was purchased from Shanghai YuanYe Biotechnology Co., Ltd. (Shanghai, China). Dextran sodium sulfate (DSS) was supplied by MP Biomedicals (Santa Ana, CA, USA). Enzyme-linked immunosorbent assay (ELISA) kits for mouse tumor necrosis factor α (TNF-α), interleukin (IL)-1β, and IL-6 were ordered from Biolegend (San Diego, CA, USA). Superoxide dismutase (SOD), malondialdehyde (MDA), catalase (CAT), and total antioxidant capacity (T-AOC) test kits were ordered from Beyotime Biotechnology Co., Ltd. (Shanghai, China). A reduced glutathione (GSH) assay kit was ordered from Nanjing Jiancheng Bioengineering Research Institute Co., Ltd. (Nanjing, China). The tissue iron content assay kit was ordered from Beijing Solarbio Science & Technology Co., Ltd. (Beijing, China).

### 2.2. Animals and Administration Protocols

A total of 36 C57BL/6 male mice (aged 7 weeks, weighing 20–22 g) were ordered from Liaoning Changsheng Biotechnology Co., Ltd. (Benxi, China) and accommodated in a rearing room with adequate water, food, and light conditions. Following normal feeding for one week, the mice were randomized into 6 groups (6 mice per group) according to the experimental conditions, namely the control group, Amy-H (high-dose, 50 mg/kg) group, DSS-treated group, DSS + Amy-L (low-dose, 12.5 mg/kg) group, DSS + Amy-M (median-dose, 25 mg/kg) group, and DSS + Amy-H (high-dose, 50 mg/kg) group. The doses used in this study were selected following the suggestions of previous reports [9]. The duration of the study was 21 d, during which time the mice in the control and DSS-treated groups were given saline daily via gavage, while the mice in the remaining groups were administered the corresponding dose of Amy by gavage daily. From day 14 onwards, except for the control group and the Amy-H group, the drinking water in the remaining groups was changed to 2.5% (*w*/*v*) DSS solution for 7 d to induce acute colitis. The colon contents were sampled for 16S rRNA sequencing. Following this, the mice were sacrificed via cervical dislocation on day 21, and colon tissues were sampled for subsequent experiments. Animal experiments were approved by the Institutional Animal Care and Use Committee of Jilin University (number of permit: SY202302023).

### 2.3. Disease Activity Index (DAI) Score

Variations in body weight, fecal hardness, and bloody stools were recorded daily and scored according to the DAI scale (Table 1). The DAI was calculated as DAI = (weight loss score + stool consistency score + rectal bleeding score)/3 [24,25].

### 2.4. Pathohistological Observation and Score of the Colon Tissues

The distal portion of the colon was collected and fixed in 4% paraformaldehyde. Then the colon was embedded in paraffin, sectioned, and stained with H&E. The score was based on the morphology of epithelial cells and the infiltration of inflammatory cells [26]. The reference scoring criteria are shown in Table 2.

### 2.5. Detection of Cytokines and Oxidative Stress-Related Indicators

The levels of TNF-α, IL-1β, and IL-6 were assayed using ELISA kits (Biolegend, San Diego, CA, USA) as described by the manufacturer, and the levels or activities of SOD, GSH, MDA, CAT, and tissue iron content were assayed using commercially available kits. Myeloperoxidase (MPO) activity was assessed as described in previous reports [27,28].

### 2.6. Transmission Electron Microscopy (TEM) Analysis

Colon tissues were subjected to fixation, dehydration, embedding, sectioning, and staining according to the standard experimental procedures. Sections were then observed using a HITACHI HT7800 TEM (Tokyo, Japan).

### 2.7. Western Blot and Immunofluorescence Staining

Total protein was collected after tissue homogenization. Proteins separated by 12% SDS-PAGE were electrotransferred to a PVDF membrane. After blocking with milk, the PVDF membrane was incubated with the primary antibody followed by treatment with the corresponding horseradish peroxidase-labeled secondary antibody. The protein bands were visualized using the electrochemiluminescence detection kit (Beyotime, Shanghai, China). Information on the antibodies employed in this study is given in Table S1. In addition, immunofluorescence staining was performed as previously reported [29].

### 2.8. Intestinal Flora Sequencing

In this study, high-throughput 16S rRNA sequencing of fecal bacteria was completed on the Illumina Miseq platform (provided by Shanghai Personal Biotechnology Co., Ltd., Shanghai, China). Non-repeat sequences were analyzed according to 97% similarity clustering of the optional taxonomic units (OTUs). The α-diversity and β-diversity indices were calculated based on OTU data. A combination of principal coordinate analysis (PCoA) and non-metric multidimensional scale (NMDS) analysis was used to compare the differences in intestinal flora structure. The differences in microbial composition were analyzed using linear discriminant analysis (LDA) effect size (LEfSe) (LDA > 3.5, *p* < 0.05). Pearson correlation analysis was performed using the Personal Genes Cloud platform (https://www.genescloud.cn, accessed on 1 October 2022).

### 2.9. Statistical Analysis of the Data

All data were subjected to analysis using GraphPad Prism 8 software (La Jolla, CA, USA), and expressed as the mean ± standard error of the mean (SEM). One-way ANOVA combined with Tukey’s post hoc test was chosen to analyze significant differences among multiple groups. *p* < 0.05 denotes a statistically significant difference.

## 3. Results

### 3.1. Amygdalin (Amy) Administration Attenuates Symptoms of DSS-Induced Colitis in Mice

**Figure 1:**
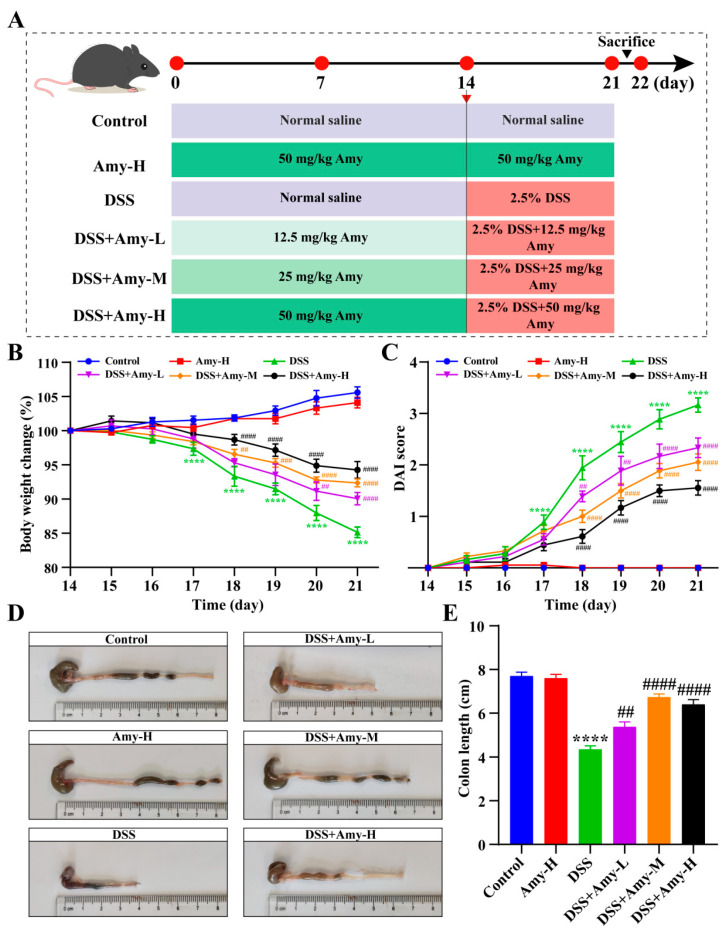
Effect of Amy administration on the symptoms of DSS-induced colitis in mice. (**A**) Experimental procedure diagram. (**B**,**C**) Body weight changes and DAI scores (*n* = 6). Colors represent different groups. (**D**) Representative photographs of the colon (*n* = 6). (**E**) Changes in colon length (*n* = 6). Data are expressed as mean ± SEM. Compared to the control group, **** *p* < 0.0001. Compared to the DSS group, ^##^ *p* < 0.01, ^###^ *p* < 0.001, and ^####^ *p* < 0.0001.

Initially, ulcerative colitis in mice was induced with 2.5% DSS (Figure 1A). Compared with mice in the control group, the mice in the DSS group exhibited excessive colonic inflammation characterized by weight loss and high disease activity index (DAI) scores, whereas different doses (12.5, 25, and 50 mg/kg) of Amy administration significantly prevented the symptoms of colitis in the mice, including increased body weight and reduced DAI scores (Figure 1B,C). Furthermore, DSS treatment led to a significant reduction in colon length (from 7.708 ± 0.18 to 4.35 ± 0.16 cm) in mice, whereas Amy administration (12.5, 25, and 50 mg/kg) significantly increased colon length in mice with colitis (Figure 1D,E). These data demonstrate that Amy administration improves colitis symptoms in mice.

### 3.2. Amy Administration Prevents DSS-Induced Colonic Pathological Damage in Mice

**Figure 2:**
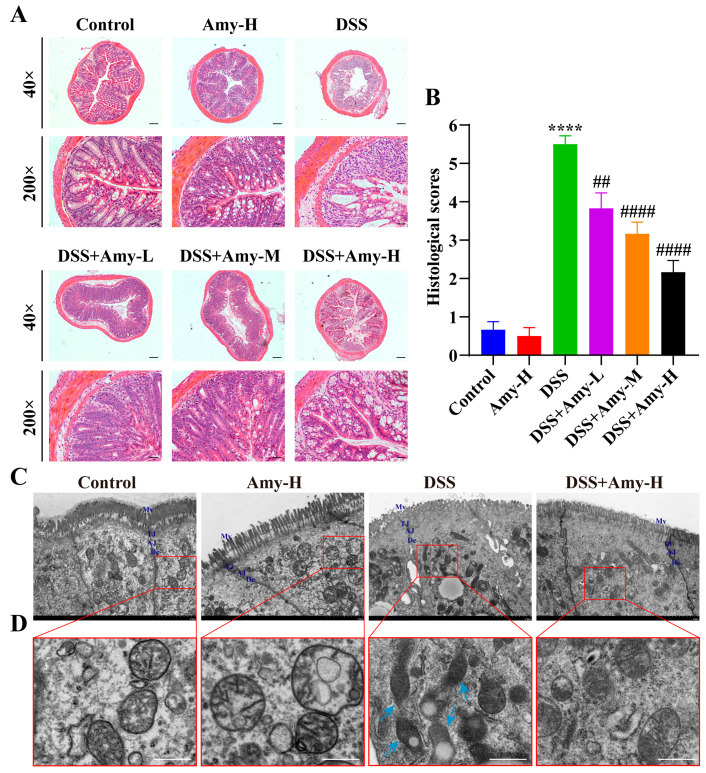
Effect of Amy administration on DSS-induced pathological damage to the colon of mice. (**A**,**B**) Representative micrographs of H&E staining and histological scoring of colon tissues (*n* = 6). Scale bar: 250 μm (top) and 50 μm (bottom). (**C**) Representative micrographs of intestinal epithelial cell ultrastructure by TEM. Scale bar: 2 μm. Mv: microvilli; TJ: tight junction; AJ: adherens junction; De: desmosomes. (**D**) Representative micrographs of mitochondria in the intestinal epithelial cell by TEM. Scale bar: 500 nm. Blue arrows show damaged and shrunken mitochondria. Compared to the control group, **** *p* < 0.0001. Compared to the DSS group, ^##^ *p* < 0.01, and ^####^ *p* < 0.0001.

H&E staining was performed to evaluate the extent of colonic mucosal damage. The mice in the control group had normal colonic histomorphology with intact mucosa and no inflammatory infiltration. In contrast, DSS treatment resulted in the loss of colonic crypts and goblet cells, pro-inflammatory cell infiltration, and elevated histological damage scores in mice (Figure 2A,B). Compared to the DSS-treated group, Amy administration to mice resulted in relatively intact colonic glands and goblet cells, less intestinal epithelial damage, and lower histological scores (Figure 2A,B), suggesting that Amy administration prevents DSS-induced colonic histopathological damage. To observe the state of the epithelial cells in colonic tissue in detail, TEM was performed. In normal colonic epithelial cells, the cells are tightly connected to each other, the mitochondria and their cristae are clearly seen in the cells, and the intestinal microvilli are numerous and well aligned. However, cell junctions (including tight junction (TJ), adherens junction (AJ), and desmosomes (De)) between the colon epithelial cells were damaged (Figure 2C), the mitochondria were shrunken and vacuolated, and the intestinal microvilli were markedly decreased in DSS-treated mice (Figure 2D). Notably, Amy administration substantially alleviated these microscopic injuries in the intestinal epithelium of colitis mice. Collectively, these results demonstrate that Amy administration can alleviate DSS-induced pathological damage to the colon.

### 3.3. Amy Administration Improves Intestinal Barrier Integrity in DSS-Treated Mice

**Figure 3:**
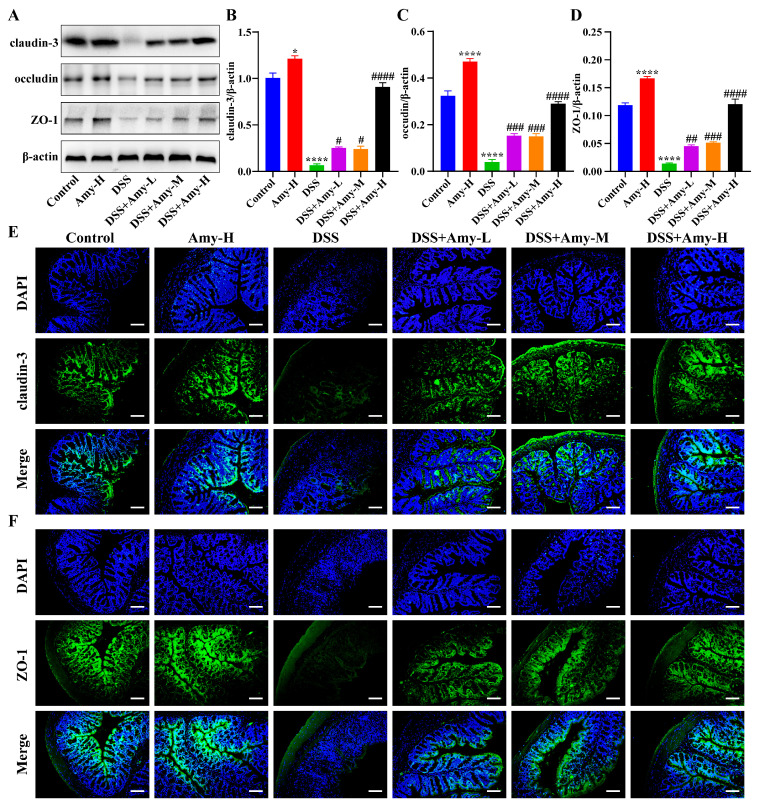
Effect of Amy administration on colonic barrier integrity in DSS-treated mice. (**A**) Representative protein bands of colonic ZO-1, claudin-3, and occludin in colon tissue. (**B**–**D**) Relative greyscale analysis of the bands (*n* = 3). (**E**,**F**) Representative immunofluorescence micrographs of colonic claudin-3 and ZO-1 (*n* = 6). Scale bar: 50 μm. All data are expressed as mean ± SEM. Compared to the control group, * *p* < 0.05, and **** *p* < 0.0001. Compared to the DSS group, ^#^ *p* < 0.05, ^##^ *p* < 0.01, ^###^ *p* < 0.001, and ^####^ *p* < 0.0001.

The disruption of the intestinal barrier is the basis for the development of IBD [30]. Members of the claudin, Zonula occludens (ZO), and occludins families are classical intercellular tight junction (TJ) proteins associated with intestinal integrity in colonic tissue [31]. In Figure 2C, we found that Amy administration improved the TJ between cells in mice with colitis. Thus, the expression of TJ-related proteins in the colon was then examined using Western blot and immunofluorescence. The Western blot results showed that DSS treatment reduced the levels of claudin-3, ZO-1, and occludin compared with treatment using the control group, while Amy administration significantly elevated the levels of claudin-3, ZO-1, and occludin in DSS-treated mice (Figure 3A–D). Moreover, the results of immunofluorescence staining further corroborated the above findings (Figure 3E,F). Collectively, these data demonstrate that Amy administration prevents DSS-induced disruption of the intestinal barrier in mice.

### 3.4. Amy Administration Reduces DSS-Induced Colitis by Modulating Excessive Colonic Inflammatory Responses

**Figure 4:**
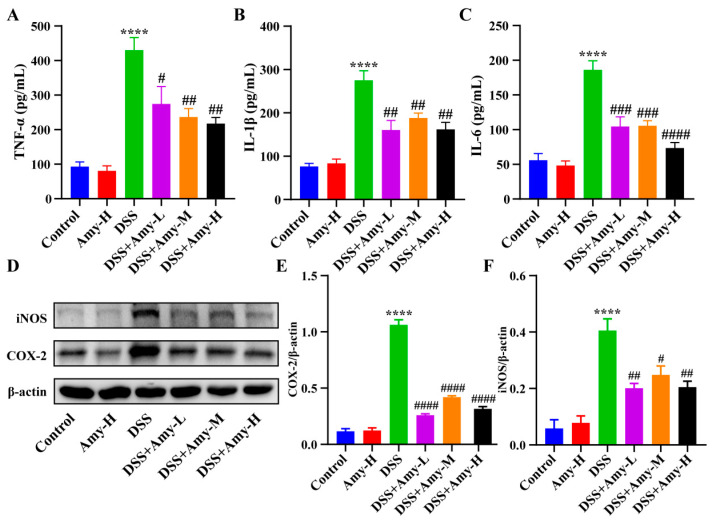
Effect of Amy administration on the levels of pro-inflammatory mediators in colon tissues of DSS-induced mice. (**A**–**C**) Levels of colonic TNF-α, IL-1β, and IL-6 (*n* = 6). (**D**–**F**) Representative protein bands of COX-2 and iNOS in colon tissues and the corresponding relative density analysis results (*n* = 3). Data are expressed as mean ± SEM. Compared to the control group, **** *p* < 0.0001. Compared to the DSS group, ^#^ *p* < 0.05, ^##^ *p* < 0.01, ^###^ *p* < 0.001, and ^####^ *p* < 0.0001.

**Figure 5:**
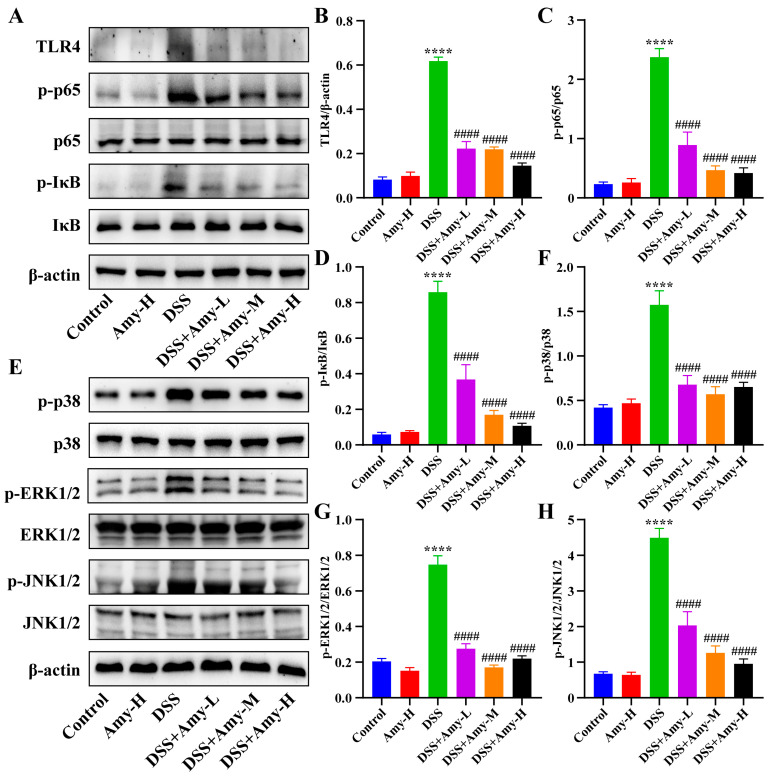
Effect of Amy administration on DSS-induced activation of TLR4-NF-κB/MAPK signaling pathways in colon tissues. (**A**) Representative protein bands of colonic TLR4, p65, p-p65, IκB, and p-IκB and (**B**–**D**) the corresponding relative density analysis results (*n* = 3). (**E**) Representative Western blot bands of colonic p38, p-p38, ERK1/2, p-ERK1/2, JNK1/2, and p-JNK1/2 and (**F**–**H**) the corresponding relative density analysis results (*n* = 3). All data are expressed as mean ± SEM. Compared to the control group, **** *p* < 0.0001. Compared to the DSS group, ^####^ *p* < 0.0001.

To evaluate the effect of Amy administration on colonic inflammation in mice, the levels of TNF-α, IL-1β, IL-6, cyclooxygenase (COX)-2, and inducible nitric oxide synthase (iNOS) were examined in the colon tissue. The results showed that DSS treatment dramatically raised the levels of TNF-α, IL-1β, IL-6, COX-2, and iNOS in the colon tissue of mice compared with that of the control group (Figure 4). However, Amy administration significantly decreased the levels of these mediators (Figure 4). In addition, the activation of important inflammation-related signaling pathways nuclear factor kappa B (NF-κB) and mitogen-activated protein kinase (MAPK) was examined (Figure 5A,E). In line with the trend of pro-inflammatory mediators, DSS treatment elevated the levels of toll-like receptor 4 (TLR4) (Figure 5B) and the phosphorylation levels of p65 (Figure 5C), IκB (Figure 5D), p38 (Figure 5F), ERK1/2 (Figure 5G), and JNK1/2 (Figure 5H), whereas Amy administration significantly decreased the levels of TLR4 and the phosphorylation levels of IκB, p65, p38, ERK1/2, and JNK1/2 (Figure 5), indicating that Amy administration prevented the activation of NF-κB and MAPK signaling pathways. Overall, these data demonstrate that Amy administration can prevent DSS-induced excessive colonic inflammatory responses.

### 3.5. Amy Administration Reduced DSS-Induced Oxidative Stress and Inhibited Intestinal Epithelial Cell Apoptosis and Ferroptosis

**Figure 6:**
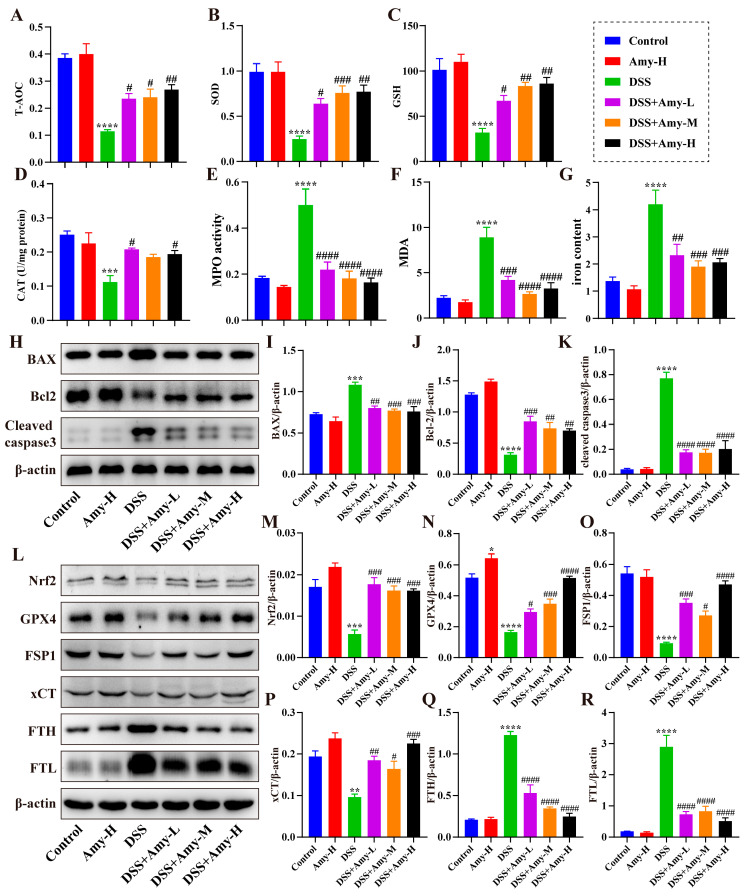
Effect of Amy administration on DSS-induced intestinal oxidative stress and intestinal epithelial cell apoptosis and ferroptosis. (**A**) T-AOC (*n* = 6). (**B**) SOD (*n* = 6). (**C**) GSH (*n* = 6). (**D**) CAT (*n* = 6). (**E**) MDA (*n* = 6). (**F**) MPO (*n* = 6). (**G**) Iron content (*n* = 6). (**H**) Representative Western blot bands of colonic BAX, Bcl-2, and cleaved caspase3. (**I**–**K**) The corresponding relative density analysis results (*n* = 3). (**L**) Representative Western blot bands of colonic Nrf2, GPX-4, FSP1, xCT, FTH, and FTL. (**M**–**R**) The corresponding relative density results (*n* = 3). All data are expressed as mean ± SEM. Compared to the control group, * *p* < 0.05, ** *p* < 0.01, *** *p* < 0.001, and **** *p* < 0.0001. Compared to the DSS group, ^#^ *p* < 0.05, ^##^ *p* < 0.01, ^###^ *p* < 0.001, and ^####^ *p* < 0.0001.

Oxidative stress is associated with the progression of IBD. The levels or activities of malondialdehyde (MDA), myeloperoxidase (MPO), total antioxidant capacity (T-AOC), superoxide dismutase (SOD), catalase (CAT), and glutathione (GSH) were determined. The results showed that the levels of T-AOC (Figure 6A), SOD (Figure 6B), GSH (Figure 6C), and CAT (Figure 6D) were decreased, and MPO activity (Figure 6E) and MDA levels (Figure 6F) were elevated in the colon tissue of DSS-treated mice compared with that of the control group. In contrast, Amy administration (12.5, 25, and 50 mg/kg) significantly reduced MDA levels and MPO activity and increased T-AOC, SOD, GSH, and CAT levels (Figure 6A–F), indicating that Amy administration reduced DSS-induced oxidative stress. In addition, the levels of apoptosis-related proteins B cell lymphoma (Bcl)-2, Bcl-2-associated X (BAX), and cleaved caspase3 (Figure 6H), and ferroptosis markers MDA, GSH, iron content, nuclear factor erythroid 2-related factor 2 (Nrf2), glutathione peroxidase 4 (GPX-4), ferroptosis suppressor protein 1 (FSP1), xCT (also known as SLC7A11), ferritin light chain (FTL), and ferritin heavy chain (FTH) (Figure 6L) were examined in colon tissues. DSS treatment markedly raised the levels of BAX (Figure 6I), cleaved caspase3 (Figure 6K), MDA (Figure 6F), iron content (Figure 6G), FTH (Figure 6Q), and FTL (Figure 6R) and decreased the levels of Bcl-2 (Figure 6J), GSH (Figure 6C), Nrf2 (Figure 6M), GPX4 (Figure 6N), FSP1 (Figure 6O), and xCT (Figure 6P), indicating that DSS treatment resulted in intestinal epithelial apoptosis and ferroptosis. However, Amy administration reversed the DSS-induced changes in these indicators (Figure 6), suggesting that Amy administration inhibited DSS-induced intestinal epithelial apoptosis and ferroptosis.

### 3.6. Amy Administration Regulates the Structure of the Intestinal Microbiota in DSS-Treated Mice

**Figure 7:**
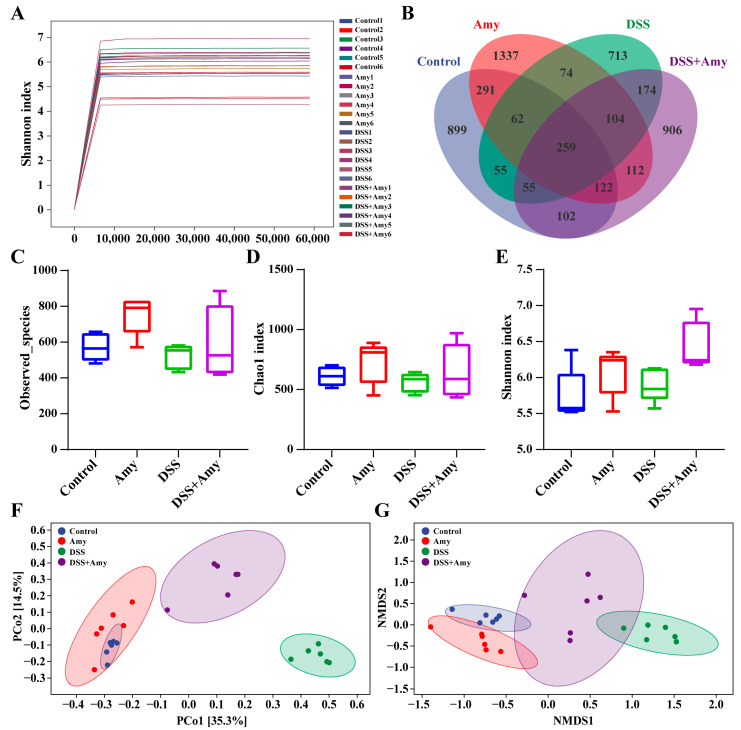
Effect of Amy administration on the diversity and structure of the intestinal flora in mice with DSS-induced colitis (*n* = 6). (**A**) Dilution curve. (**B**) Wayne diagram. (**C**) Observed_species index analysis. (**D**) Chao1 index analysis. (**E**) Shannon index analysis. (**F**) Principal coordinate analysis (PCoA). (**G**) Non-metric multidimensional scale (NMDS) analysis. OTUs: operational taxonomic units.

To determine whether Amy administration altered the intestinal flora, the colon contents of mice were subjected to 16S rRNA high-throughput sequencing. We focused on the Amy-H group when sequencing because of its better effect in terms of reducing colitis. The dilution curves, produced based on the Shannon diversity index, showed that an adequate quantity of data was captured in the present study (Figure 7A). The Venn diagram shows that 899, 1337, 713, and 906 unique OTUs were detected in the control, Amy-H, DSS, and DSS + Amy-H groups (Figure 7B), respectively. To determine the effect of Amy on the abundance and diversity of the intestinal microbiota, α-diversity, and β-diversity were evaluated in four groups of fecal samples. Different OTU-based indicators, including Observed_species (Figure 7C), Chao1 (Figure 7D), and Shannon (Figure 7E), showed that α-diversity was not affected by the oral administration of Amy. Principal coordinates analysis (PCoA) (Figure 7F) and NSMD (Figure 7G) based on Bray–Curtis distances showed that there was a separation in the intestinal flora structure between the normal control and colitis mice, indicating that these samples differed in their compositional structure. The intestinal flora structure also differed between the DSS and DSS + Amy-H groups, and the bacterial composition of the DSS + Amy-H group was closer to that of the control group (Figure 7F,G). These results suggest that oral Amy administration modulates the structure of intestinal flora in mice with colitis.

### 3.7. Amy Administration Prevents DSS-Induced Disorders of the Intestinal Flora

**Figure 8:**
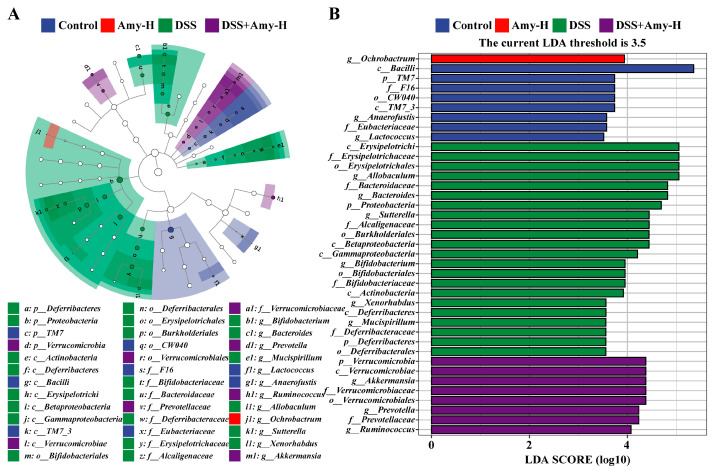
Effect of Amy administration on the composition of the gut microbiota in mice with DSS-induced colitis (*n* = 6). (**A**) Taxonomic cladogram (phylum, class, order, family and genus) from LEfSe showing taxonomic associations between groups of microbial communities. Each node represents a specific taxonomic type. Blue, red, green, and purple nodes indicate taxonomic types that are more abundant in the Control, Amy-H, DSS, and DSS + Amy-H groups than in the other groups, respectively. (**B**) LDA for the LEfSe analysis (LDA > 3.5, *p* < 0.05).

**Figure 9:**
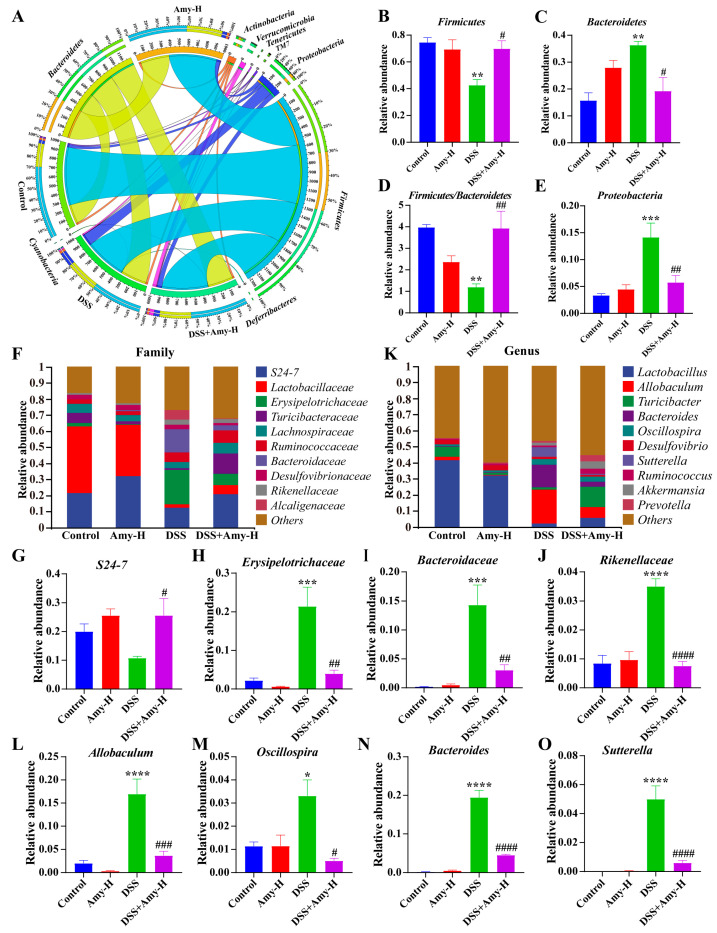
Effect of Amy administration on the relative abundance of specific intestinal bacteria in DSS-treated mice. (**A**) Circos of the intestinal flora distribution at the phylum level (*n* = 6). (**B**–**E**) Differences in the relative abundance of intestinal bacteria at the phylum level (*n* = 6). (**F**) The top 10 intestinal bacteria in relative abundance at the family level (*n* = 6). (**G**–**J**) Differences in the relative abundance of specific intestinal bacteria at the family level (*n* = 6). (**K**) The top 10 intestinal bacteria in relative abundance at the genus level (*n* = 6). (**L**–**O**) Differences in the relative abundance of specific intestinal bacteria at the genus level (*n* = 6). All data are expressed as mean ± SEM. Compared to the control group, * *p* < 0.05, ** *p* < 0.01, *** *p* < 0.001, and **** *p* < 0.0001. Compared to the DSS group, ^#^ *p* < 0.05, ^##^ *p* < 0.01, ^###^ *p* < 0.001, and ^####^ *p* < 0.0001.

**Figure 10:**
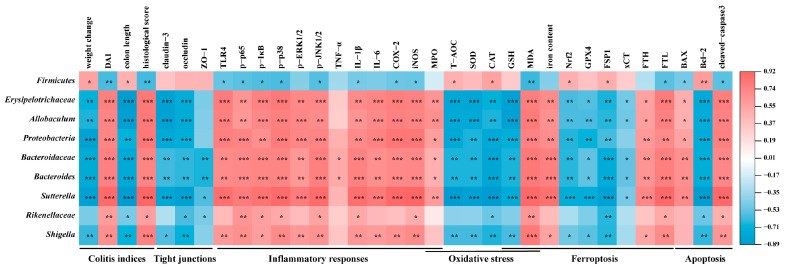
Spearman correlation analysis between intestinal flora and colitis-related indices. (* *p* < 0.05, ** *p* < 0.01, and *** *p* < 0.001).

Next, the significant differences in gut flora among different groups were assessed through LEfSe. The results showed that *Anaerofustis* and *Lactococcus* were the dominant bacteria in the control group and *Ochrobactrum* was dominant in the Amy-H group. *Allobaculum*, *Bacteroides*, and *Sutterella* were the dominant bacteria in the DSS group, while *Akkermansia*, *Prevotella,* and *Ruminococcus* were identified as the dominant bacteria in the DSS + Amy-H group (Figure 8). Following this, variations in the mouse intestinal flora at different taxonomic levels were analyzed. At the phylum level (Figure 9A), DSS treatment decreased the relative abundance of *Firmicutes* (Figure 9B), increased the relative abundance of *Bacteroidetes* (Figure 9C) and *Proteobacteria* (Figure 9E), and raised the ratio of *Firmicutes* to *Bacteroidetes* (Figure 9D). However, Amy administration nearly reversed these changes. At the family level (Figure 9F), Amy administration significantly increased the relative abundance of *S24-7* (Figure 9G) and decreased the relative abundance of *Erysipelotrichaceae* (Figure 9H), *Bacteroidaceae* (Figure 9I), and *Rikenellaceae* (Figure 9J) in the DSS-treated mice. At the genus level (Figure 9K), Amy administration lowered the relative abundance of *Allobaculum* (Figure 9L), *Oscillospira* (Figure 9M), *Bacteroides* (Figure 9N), and *Sutterella* (Figure 9O) in the DSS-treated mice. Finally, Spearman correlation analysis was employed to identify the gut bacteria associated with colitis parameters like TJ, inflammatory response, oxidative stress, and cell death (Figure 10). The results showed that specific microorganisms were positively correlated with colitis symptoms, pathological damage, inflammatory response, oxidative stress, and cell death, and negatively correlated associated with body weight, colon length, and TJ. For example, *Erysipelotrichaceae*, *Bacteroidaceae*, *Allobaculum*, *Bacteroides*, and *Sutterella* were positively correlated with DAI, inflammatory response, MDA, iron content, FTH, FTL, and cleaved caspase3, but negatively correlated with body weight, colon length, TJ, antioxidant indices (T-AOC, SOD, CAT, and GSH), Nrf2, GPX4, FSP1, and BCL2. The above results suggest that the improving effect of Amy on colitis is closely related to the regulation of the intestinal microbiome.

## 4. Discussion

Inflammatory bowel disease (IBD) is classified into ulcerative colitis (UC) and Crohn’s disease (CD). Its pathogenesis is very complex, being associated with complex interplay the host immune response, genetic susceptibility, gut microbiota, and environmental conditions [32]. Currently, IBD is incurable and recurrent, seriously affecting the life quality of patients. The drugs available for IBD treatment have poor effectiveness [4]. Therefore, there is a great need and urgency to discover novel drugs for IBD [33]. Amygdalin (Amy) exerts anti-inflammatory and antioxidant effects in multiple diseases [34]. In the present study, Amy administration alleviated colitis symptoms (as manifested by decreased weight loss, increased colon length, and reduced DAI scores) and colonic tissue damage in mice with colitis by suppressing DSS-induced colonic inflammatory responses, oxidative stress, and epithelial cell death as well as rebalancing intestinal flora, demonstrating that Amy may serve as a preventive and therapeutic agent for IBD.

The intestinal barrier is an imperative safeguard against invasion by pathogenic bacteria and foreign bodies, and its disruption underlies the development of IBD [30]. Thus, preserving intestinal barrier integrity can be crucial to the prevention and treatment of IBD. The intestinal epithelial barrier is mainly composed of intestinal epithelial cells and intercellular tight junction (TJ) proteins (e.g., claudin-3, occludin, and ZO-1) that regulate the permeability of the barrier [31]. Studies have shown that the levels of colonic TJ proteins are significantly reduced in IBD patients and mice with UC, leading to increased intestinal permeability [35,36]. Consistent with these reports, in the present study, transmission electron microscopy results showed that DSS treatment disrupted TJ between intestinal epithelial cells, whereas Amy administration improved TJ. Furthermore, both Western blot and immunofluorescence results showed that DSS treatment resulted in significantly lower levels of claudin-3, occludin, and ZO-1 proteins in the colon tissues compared with those of the control group, while Amy administration restored the levels of these proteins remarkably. These results suggest that the relieving effect of Amy against colitis in mice correlates with an improvement in the intestinal barrier.

The process of IBD is accompanied by an inflammatory cascade and the release of multiple pro-inflammatory mediators. High levels of pro-inflammatory mediators trigger intestinal inflammation. The cytokines tumor necrosis factor-α (TNF-α), interleukin (IL)-6, and IL-1β, as well as the enzymes COX-2 and iNOS, are important mediators of the colonic inflammatory response [37]. In the present study, levels of colonic COX-2, iNOS, TNF-α, IL-1β, and IL-6 were markedly elevated in DSS-treated mice compared with those of the control group, while Amy administration markedly lowered the levels of these pro-inflammatory mediators. Surprisingly, a very recent article found that essential oil from Pruni Semen (PSEO), whose main components are products of Amy hydrolysis, had a protective effect against DSS-induced ulcerative colitis in mice, markedly ameliorating the symptoms of colitis, attenuating histopathological damage, and diminishing the production of pro-inflammatory cytokines [11]. This is consistent with the results of the present study and further supports the anti-colitis effect of Amy. In addition, to further explore the mechanisms by which Amy attenuated colonic inflammation, we investigated the changes in inflammatory signaling pathways after Amy administration. Toll-like receptor 4 (TLR4) is the most well-characterized member of the TLR family, which recognizes pathogen-associated molecular patterns, like lipopolysaccharide (LPS), promotes cytokines production via downstream signaling, and plays an essential role in innate immune and inflammatory responses [38]. The nuclear factor kappa B (NF-κB) and mitogen-activated protein kinase (MAPK) signaling pathways are important signaling transducers downstream of TLR4 [39,40] and studies show that TLR4 is significantly increased in UC patients [41], while reduced NF-κB and MAPK signaling pathway activation is associated with the remission of colitis in mice [42,43]. In the present study, colonic TLR4 expression, phosphorylation levels of NF-κB (p65 and IκB), and MAPK (p38, ERK1/2, and JNK1/2) signaling pathway proteins were markedly elevated in mice with colitis compared with the control group, while Amy administration markedly decreased the TLR4 expression and phosphorylation levels of these pathway proteins. These results suggest that Amy administration suppresses inflammation-related signaling (TLR4-NF-κB/MAPK) and reduces the production of pro-inflammatory mediators, thereby providing relief from colonic inflammation.

In addition, oxidative stress correlates strongly with the development of inflammation and directly triggers the release of various cytokines (e.g., IL-6, IL-1β, and TNF-α), which are also considered to be among the potential causative factors of IBD [44,45]. Malondialdehyde (MDA), a product of lipid peroxidation, is a well-recognized marker of excessive oxidative stress [46]. Myeloperoxidase (MPO), a marker of tissue damage and neutrophil infiltration [47], is remarkably elevated in the colon tissue of IBD patients and DSS-challenged mice [48]. The body’s antioxidant system is largely dependent on superoxide dismutase (SOD), catalase (CAT), and glutathione (GSH). To confirm the effect of Amy administration on oxidative stress in DSS-treated mice, we measured the levels or activities of total antioxidant capacity (T-AOC), MDA, MPO, CAT, GSH, and SOD in colon tissues. Intriguingly, Amy administration (12.5, 25, and 50 mg/kg) markedly decreased MDA levels and MPO activity and increased the activities of CAT and SOD, as well as the levels of GSH and T-AOC, in the colon of DSS-treated mice, indicating that Amy administration was capable of reducing DSS-induced colonic oxidative stress.

More importantly, inflammation and oxidative stress can lead to cellular damage and even death. Because the intestinal barrier is disrupted, apoptosis is almost inevitable as colitis progresses [49]. Therefore, we examined the expression of apoptosis-related proteins (BAX, Bcl-2, and cleaved caspase3) in the colon. The results showed that Amy administration significantly inhibited BAX and cleaved caspase3 expression in the colon, while promoting Bcl-2 expression, indicating that Amy administration could reduce DSS-triggered apoptosis in the intestinal epithelium. Furthermore, ferroptosis, an iron-dependent cell-regulated cause of death identified in recent years, was found to be present in the colon tissue of DSS-treated mice and patients with IBD, and inhibition of ferroptosis improved colitis symptoms in mice [50], suggesting that the suppression of ferroptosis could be a potential therapeutic option for IBD. Moreover, Amy can reduce inflammation and oxidative responses by promoting ferroptosis-related protein expression such as GPX-4 and Nrf2 [19], hinting at a potential link to ferroptosis. Therefore, we examined the effects of Amy administration on ferroptosis. The levels of MDA, iron content, FTH, and FTL were increased, and the levels of GSH, Nrf2, GPX4, FSP1, and xCT were decreased in the colon tissue of DSS-treated mice compared with that of the control group, while Amy administration reversed the changes of these ferroptosis markers in colon tissues, indicating that Amy administration suppressed ferroptosis in the intestinal epithelium of mice. Based on the above results, we speculate that the reduction in intestinal epithelial apoptosis and ferroptosis observed in mice with colitis may be a result of the improvement of intestinal inflammation and oxidative stress by Amy treatment, but the regulatory relationship remains to be further investigated.

Growing quantities of research into humans and mice demonstrate that intestinal flora disorders contribute to the progression of IBD [20]. An imbalance of intestinal flora can cause gut epithelial dysfunction and mucosal lesions [21]. For example, harmful substances produced by the intestinal flora, such as lipopolysaccharides (LPS) produced by Gram-negative bacteria, can be recognized by TLR4, triggering inflammatory responses and oxidative stress, etc. In turn, the enhanced inflammatory response exacerbates the defective intestinal barrier, leading to colonic leakage and thus exacerbating IBD [51]. Via gut flora sequencing, we showed that Amy administration significantly improved the structure of intestinal flora in mice with colitis, making its structure resemble that of normal mice in the control group. Moreover, Amy administration adjusted the composition of the intestinal flora. Specifically, at the phylum level, Amy selectively reduced the abundance of *Bacteroidetes* and *Proteobacteria* and raised the abundance of *Firmicutes* and the ratio of *Firmicutes* to *Bacteroidetes* in DSS-treated mice. *Firmicutes*, together with *Bacteroidetes*, are the predominant phyla comprising the gut microflora. A clinical study showed that the ratio of *Firmicutes* to *Bacteroides* (F/B) was decreased in patients with UC [52], whereas the abundance of *Proteobacteria*, a hallmark feature of intestinal dysbiosis, was markedly elevated in mice with colitis [53]. At the family level, Amy administration significantly raised the relative abundance of *S24-7* in DSS-treated mice, which is known to be capable of degrading complex carbohydrates, and this correlated with the relief of colitis [54]. In addition, we observed a marked rise in the abundance of pathogenic bacteria like *Erysipelotrichaceae*, *Rikenellaceae*, and *Bacteroidaceae* in mice with colitis. *Erysipelotrichaceae* was reported to be in high abundance in colorectal cancer [55]. *Rikenellaceae* and *Bacteroidaceae* were associated with inflammation and were in elevated abundance in mice with colitis [56,57]. Notably, Amy administration markedly reduced the abundance of these bacteria in mice with colitis. At the genus level, DSS treatment raised the abundance of *Allobaculum* [58], *Oscillospira* [59], *Bacteroides* [60], and *Sutterella* [61] in the gut, all of which are associated with colitis or colon cancer. *Oscillospira*, for example, is linked to colon cancer [59]. *Bacteroides* can disrupt the intestinal barrier and its metabolite succinic acid can boost intestinal inflammation in patients with IBD [60]. Interestingly, Amy administration markedly lowered the abundance of these bacteria in mice with colitis. Notably, LEfSe showed that *Ochrobactrum* was enriched in the Amy-H group. This has previously been reported to be abnormally elevated during UC exacerbation [62], but its exact role requires further investigation. The above results indicate that the protective effect of Amy is closely associated with the inhibition of DSS-triggered alterations in the gut flora. Notably, studies have shown that intestinal flora metabolites are involved in the regulation of ferroptosis. For example, short-chain fatty acids are associated with DSS-induced ferroptosis in colon tissue [63]. The intestinal flora metabolite glycochenodeoxycholate promotes ferroptosis [64], while capsiate inhibits ferroptosis [65,66]. In the present study, we found that *Allobaculum*, *Bacteroides*, and *Sutterella* were positively correlated with pro-ferroptosis indicators such as iron content, FTH, and FTL, but negatively correlated with anti-ferroptosis indicators such as GSH, Nrf2, GPX4, and FSP1, suggesting that the intestinal flora may be involved in the regulation of ferroptosis by Amy. However, given the limitations of 16S rRNA sequencing, the exact association of gut microbiota changes at the species level with colitis and ferroptosis requires further investigation.

## 5. Conclusions

In summary, Amy administration reduced colitis by regulating intestinal flora, decreasing the inflammatory response and oxidative stress in the intestine, and inhibiting intestinal epithelial apoptosis and ferroptosis, thereby reducing intestinal tissue damage. Furthermore, molecular mechanism studies revealed that the downregulation of the TLR4-MAPK/NF-κB signaling cascade may be closely related to the remission of colitis. The present study may offer novel insights and alternatives for the management of IBD.

## Figures and Tables

**Table 1:** DAI score criteria.

Score	Weight Loss (%)	Stool Consistency	Rectal Bleeding
0	0	normal	none
1	1–3	meager stool	local bleeding
2	3–6		
3	6–9		
4	≥9	watery diarrhea	massive bleeding

**Table 2:** Histology scoring criteria.

Score	Degree of Epithelial Damage	Degree of Infiltration
0	normal	none
1	loss of goblet cells	infiltration surrounding crypt basis
2	loss of goblet cells in large areas	infiltration into the mucosa
3	loss of crypts	widespread infiltration into the mucosa
4	loss of crypts in large areas	infiltration into the submucosa

## Data Availability

All data needed are present in the paper.

## Supplementary Materials

The following supporting information can be downloaded at: https://www.mdpi.com/article/10.3390/cells13050444/s1, Table S1: Information on the antibodies used in this study.
