# Supplementary material for: Amygdalin Alleviates DSS-Induced Colitis by Restricting Cell Death and Inflammatory Response, Maintaining the Intestinal Barrier, and Modulating Intestinal Flora
Source: Cells. 2024 Mar 3;13(5):444. doi: 10.3390/cells13050444 (PMC10931221; doi:10.3390/cells13050444)
Supplement: Supplementary file 1 [file cells-13-00444-s001.zip › cells-2854801-supplementary.pdf]

Table S1. Information on the antibodies used in this study.

Antibody	Manufacturers	Catalog number	Dilution ratio
BAX	Proteintech	50599-2-Ig	1: 2000
β-actin	Proteintech	66009-1-Ig	1: 8000
Bcl-2	Proteintech	68103-1-Ig	1: 2000
caspase3	Cell Signaling Technology	#9662	1: 1000
claudin-3	Abcam	ab15102	1: 4000
COX-2	Proteintech	66351-1-Ig	1: 4000
ERK1/2	Proteintech	11257-1-AP	1: 4000
FSP1	Proteintech	20886-1-AP	1: 2000
FTH	Santa Cruz Biotechnology	sc-376594	1: 2000
FTL	Santa Cruz Biotechnology	sc-74513	1: 2000
GPX4	Santa Cruz Biotechnology	sc-166570	1: 2000
IκB	Proteintech	10268-1-AP	1: 2000
iNOS	Proteintech	18985-1-AP	1: 800
JNK1/2	Cell Signaling Technology	#9252	1: 1000
Nrf2	Proteintech	16396-1-AP	1: 2000

occludin	Proteintech	27260-1-AP	1: 2000
p38	Cell Signaling Technology	#8690	1: 2000
p65	Cell Signaling Technology	#8242	1: 1000
p-ERK1/2	Cell Signaling Technology	#4370	1: 1000
p-IκB	Cell Signaling Technology	#2859	1: 800
p-JNK1/2	Cell Signaling Technology	#9251	1: 500
p-p38	Santa Cruz Biotechnology	sc-166182	1: 2000
p-p65	Cell Signaling Technology	#3033	1: 1000
TLR4	Santa Cruz Biotechnology	sc-293072	1: 200
xCT	Proteintech	26864-1-AP	1: 1000
ZO-1	Proteintech	21773-1-AP	1: 4000
HRP-Conjugated AffiniPure Goat Anti-mouse IgG (H+L)	Bosterbio	BA1051	1: 6000
HRP Conjugated AffiniPure Goat Anti-rabbit IgG (H+L)	Bosterbio	BA1055	1: 6000
